# Progressive Disruption of Sphingosine-1-Phosphate Receptor 1 Correlates with Blood-Brain Barrier Leakage in A Rat Model of Chronic Hypoxic Hypoperfusion

**DOI:** 10.14336/AD.2024.0098

**Published:** 2024-05-21

**Authors:** Jeffrey Thompson, Yirong Yang, Kelsey Duval, Michael Griego, Haojie Chen, Karen SantaCruz, Haoran Deng, Carlos Perez, Sean Maez, Sasha Hobson, Theodore Li, Halima Akter, Michel Torbey, Yi Yang

**Affiliations:** ^1^Department of Neurology,; ^2^Memory and Aging Center,; ^3^College of Pharmacy,; ^4^Department of Pathology, University of New Mexico School of Medicine, Albuquerque, NM 87111, USA

**Keywords:** hypoxic hypoperfusion, Sphingosine-1-phosphate receptor 1, endothelial dysfunction, BBB permeability, white and grey matter lesion, small vessel disease

## Abstract

Endothelial dysfunction and blood-brain barrier (BBB) leakage have been suggested as a fundamental role in the development of cerebral small vessel disease (SVD) pathology. However, the molecular and cellular mechanisms that link cerebral hypoxic hypoperfusion and BBB disruption remain elusive. Sphingosine-1-phosphate (S1P) regulates the BBB integrity by binding to its receptor isoform 1 (S1PR_1_) on endothelial cells. This study tested the hypothesis that hypoxic hypoperfusion triggers capillary endothelial S1PR_1_ disruption, which compromises BBB integrity and leads to SVD-related neuropathological changes, using a chronic hypoxic hypoperfusion model with BBB dysfunction. Spontaneously hypertensive rat stroke-prone underwent unilateral carotid artery occlusion (UCAO) followed by a Japanese permissive diet (JPD) for up to 9 weeks. Selective S1PR_1_ agonist SEW2871 was used to activate S1PR_1_. Significant progressive reduction of S1PR_1_ was detected in rat brains from 4 to 9 weeks following UCAO/JPD onset, which was also detected in cerebral vasculature in human SVD. S1PR_1_ activation by SEW2871 significantly reduced lesions in both white and grey matter and ameliorated cerebral blood flow. SEW2871 reversed the loss of endothelial S1PR_1_ and tight junction proteins, and significantly attenuated UCAO/JPD induced accumulation of neuronal phosphorylated tau. This protective role of SEW2871 is associated with promotion of Akt phosphorylation and inhibition of S1PR_2_/Erk1/2 activation. Our data suggest S1PR_1_ signalling as a potential molecular mechanistic basis that links hypoxic hypoperfusion with BBB damage in the neuropathological cascades in SVD. The reversal of BBB disruption through pharmacological intervention of S1PR_1_ signalling likely reveals a novel therapeutic target for SVD.

## INTRODUCTION

Cerebral small vessel disease (SVD), the major form of vascular contributions to cognitive impairment and dementia (VCID), accounts for approximately 25-30% of ischemic strokes and contributes to 50% of all dementias and functional loss worldwide [1, 2]. SVD involves vascular pathological processes in the arteries, capillaries and small veins of the brain under a hypoxic hypoperfusion condition resulting from hypertension, diabetes, and hyperlipidemia [1, 3-7]. Once initiated, SVD progresses slowly but inevitably toward its devastating final consequences. Currently, the prevention of onset or progression of SVD is hampered by a lack of knowledge of the molecular and cellular mechanisms that underlie its basic vascular pathobiology [8].

Clinical and neuroimaging studies have suggested a fundamental role of blood-brain barrier (BBB) leakage in the progressive development of SVD pathology [9-13]. The BBB is formed by endothelial cells (ECs) lining brain microvessels under the inductive influence of neighboring cell types within the neurovascular unit (NVU), including neurons, astrocytes, pericytes, microglia, and other components of the brain parenchyma that interact with ECs [14, 15]. Tight junction proteins (TJPs), the integral transmembrane proteins that form the tight junction (TJ) strands between ECs, play a key role in regulating BBB permeability [16, 17]. The BBB is a key mediator of cerebral homeostasis. Failure of the BBB plays an important role in lacunar stroke, generation of inflammatory white matter lesions (WMLs), leukoaraiosis, and other pathological features of cerebral SVD [10, 18]. Studies reported that BBB leakage correlates with WML volume and cognitive dysfunction [19], and intracerebral hemorrhage [11] in SVD. A study on long-term BBB permeability changes in patients with SVD showed increased BBB permeability in normal appearing WM surrounding the WML borders [20], suggesting that BBB disruption is associated with formation of new WMLs. Hence, it would be expected that reversal of BBB damage will accompany reduced SVD pathology. However, there is a lack of basic understanding of how cerebral hypoxic hypoperfusion is linked to morphological and molecular changes that compromise the BBB integrity.

Capillary dysfunction has been suggested to share a basic aspect in the pathogenesis of SVD [21-23]. Disruption of endothelial signalling leads to an extreme degree of capillary dysfunction and is highly conductive to BBB breakdown [21], resulting in perivascular inflammation and astrogliosis in SVD. Sphingosine-1-phosphate (S1P) is a membrane-derived signalling sphingolipid [24, 25]. S1P regulates the integrity of the BBB by binding to its receptor isoforms (S1PR_s_) on ECs and astrocytes. Of the five S1P receptors, S1PR_1,2,3_ are expressed in ECs and S1P-induced changes in BBB function are mediated through S1PR_1_ and S1PR_2_ [26]. S1P activation of S1PR_1_ enhances brain endothelial TJs and adherens junctions (AJs), reduces BBB permeability, limits leukocyte infiltration, and inhibits astrogliosis [27-29]. S1PR_2_ plays pivotal roles in CNS inflammation and increases both BBB permeability and leukocyte entry [27, 30-33]. Using a spontaneously hypertensive rat stroke-prone (SHRSP) model of SVD, we previously showed that the SHRSPs that underwent unilateral carotid artery occlusion (UCAO) followed by Japanese permissive diet (JPD) developed BBB leakage and disruption of endothelial TJs [34], leading to WMLs, neuroinflammation, and cognitive impairment, which were secondary to tissue hypoxic hypoperfusion [35-38]. Despite the prominent role played by S1PR_1_ on vascular barrier function, its effect on BBB dysfunction in SVD has not been examined. Whether endothelial S1PR_1_ is a potential link between BBB dysfunction and the tissue hypoxic hypoperfusion that underlies the essentials of SVD remains an open question.

In this study, we tested the hypothesis that chronic hypoxic hypoperfusion downregulates capillary endothelial S1PR_1_ and disrupts endothelial TJs, leading to BBB dysfunction in the SVD model of UCAO/JPD SHRSPs. We found progressive disruption of S1PR_1_ in lesioned brains of SHRSPs from 4 to 9 weeks after UCAO/JPD onset, which correlated with extensive inflammation seen at the same time-points. This decrease of S1PR_1_ expression was also detected in human brains of SVD. The timeline of the significant decrease of S1PR_1_ is consistent with an accumulation of pTau in the cortex and corpus callosum and increased expression of S1PR_2_ seen at 9 weeks. Using a S1PR_1_-selective agonist, SEW2871 [39], we further tested the potential that interventions selectively targeting S1PR_1_ signalling pathways could ameliorate the pathological progression of SVD through protection of BBB integrity. Here, we report that chronic hypoxic hypoperfusion triggers progressive disruption of S1P-S1PR_1_ signalling leading to endothelial injury and BBB dysfunction in SVD.

## MATERIALS AND METHODS

### Animal groups and surgery

All animal studies were reviewed and approved by the Institutional Animal Care and Use Committee at the University of New Mexico in accordance with institutional guidelines and conformed to the National Institutes of Health guidelines for use of laboratory animals in research. Every effort was made to minimize the number of experimental animals used and their suffering.

The methods for the unilateral carotid artery occlusion (UCAO) surgery and Japanese permissive diet (JPD) have been reported previously and will be described briefly [35]. In this study, male SHRSPs (Charles River Laboratories) were divided into the following groups: 1) Sham with normal rat chow; 2) UCAO/JPD + vehicle (DMSO + solutol); 3) UCAO/JPD + SEW2871 (0.5 mg/kg in vehicle). Rats were sacrificed at 4 weeks or 9 weeks following UCAO/JPD. Animals were purchased at 6 weeks of age and monitored with weekly weight measurements. The diet was given and UCAO was performed at 12-week of age in SHRSP. In the UCAO groups, the right carotid artery was isolated and double-ligated permanently with 5-0 silk sutures under deep anaesthesia with 2.0% isoflurane. Following UCAO, rats were placed on the JPD (16% protein, 0.75% potassium, 4% sodium; Ziegler Bros, Inc.) with 1% sodium chloride added to drinking water. In the sham group, rats were fed with regular rodent diet and tap water. The vehicle for the SEW2871 study consisted of 30% DMSO with 25% solutol in normal saline. DMSO was needed to dissolve the drug; both drug or vehicle injections were delivered intraperitoneally every other day for up to 4 weeks, beginning at 12 weeks of age.

### Autopsy brain specimens of SVD patients

Unstained sections from existing autopsy paraffin embedded tissue blocks were obtained from the University of New Mexico, Office of the Medical Investigator (OMI). Patients with the words ‘hypertension’ or ‘arteriolosclerosis’ were included. The clinical information was reviewed, and clinical diagnoses were made. Patients should be older than 40 with no upper age limit. Brain sections from these cases were screened by neuropathologist (Dr. Karen SantaCruz) for appropriate SVD/VCID pathologies (arteriolosclerosis, lacunes, and white matter rarefaction). Blocks were collected from multiple brain areas (frontal watershed, middle frontal gyrus, striatum, basal ganglia, and hippocampus) with a particular emphasis on the sites of SVD/VCID pathology in white matter (WM) and grey matter (GM). Blocks with healthy brain areas (occipital lobe) from these cases were used as non-pathological controls. Sections with healthy brain areas were selected by the neuropathologist to show no histopathological features diagnostic of a specific disease, including stains of H&E, CD68 for active microglia/macrophages, and p62 for active autophagy. Total 11 patients with 7 females (age 56-72), 3 males (age 52-59), and 1 de-identified due to the OMI database that has the information being no longer in use.

### Magnetic Resonance Imaging (MRI)

Multimodal MRI of the rat, including relaxation time imaging, diffusion imaging, perfusion imaging and dynamic permeability imaging, was conducted as described before [40]. The rat was placed in a dedicated holder and positioned in the isocenter of a 4.7-Tesla MRI scanner (Bruker BioSpin), which was equipped with a 40-cm bore, a 660 mT/m (rise time within 120 µs) gradient and shim systems. To obtain a good signal-to-noise ratio, a small-bore linear RF coil (Inner Diameter = 72 mm) and a single tuned surface coil (RAPID Biomedical) were employed for signal excitation and detection, respectively [41-43]. During MRI, rats were anaesthetized with 2.5% isoflurane by mechanical ventilation. Respiration and heart rate were monitored during MRI measurements, and body temperature was maintained at 37.0 ± 0.5 °C.

T2-weighted images were acquired with a fast spin-echo sequence (RARE) (TR/TE = 5000 ms/56 ms, FOV = 4 cm x 4 cm, slice thickness = 1 mm, interslice distance = 1.1 mm, number of slices = 12, matrix = 256 x 256, number of averages = 3). Hyperintensity lesion areas were manually delineated from T2 images. The delineated areas were used as reference for all the other parametric images. The same slice location was prescribed for all subsequent MR protocols.

Cerebral blood flow (CBF) was measured using the arterial spin labelling (ASL) method. The sequence: Flow-sensitive Alternating Inversion Recovery Rapid Acquisition with Relaxation Enhancement (FAIR-RARE) was used to implement ASL with parameters: TE/TR = 46 ms/16000 ms, FOV = 4 cm x 4 cm, slice thickness = 1 mm, number of slices = 1, matrix = 128 x 128. The perfusion map was calculated using ASL Perfusion Processing macro in ParaVision 5.1.

To non-invasively evaluate BBB permeability, we applied dynamic contrast-enhanced MRI (DCE-MRI) and graphical analysis of the resultant image data [44]. The contrast agent Gd-DTPA at a dose of 0.1 mM/kg was injected into the femoral vein. DCEMRI was performed using a transverse fast T1 mapping that consisted of obtaining precontrast (3 sequences) and postcontrast (16 sequences) images up to 45 minutes after the contrast injection. Previous research [45] has demonstrated that the blood-to-tissue transfer or influx constant, *K*_i_, could be obtained by graphical analysis of a timed series of tissue and arterial concentrations of contrast agent. Since the contrast agent concentration is proportional to changes of 1/T1(Δ(1/T1(t))), the map of *K*_i_ was constructed from repeated estimates of Δ(1/T1(t)). An in-house computer program in MATLAB (Mathworks), which implemented the principle above, was used to generate the *K*_i_ map.

### Hematoxylin and Eosin (H&E) staining

Paraffin-embedded brain sections were assessed for blood vessel changes by H&E staining using standard techniques employing Lillie’s variant of Mayer’s hemalum (Lillie- Mayer) and eosin/phloxine. Briefly, tissues were rehydrated through a graded alcohol series and exposed to hematoxylin for nuclear staining. Slides were rinsed in tap water and differentiated using acid alcohol and Scott’s tap water substitute. Eosin/phloxine counterstaining was performed followed by serial alcohol dehydration, clearing with xylenes, and coverslipping with DPX. Stained slides were viewed and imaged using an Olympus BX-51 (Center Valley, PA, USA) bright field microscope equipped with an Optronics digital camera.

### Immunohistochemistry (IHC)

Ten µm sections from rat brain fixed with 2% paraformaldehyde, 0.1 M sodium periodate, 0.075 M lysine in 100 mM phosphate buffer, pH 7.3 (PLP) were used for immunohistochemical analysis. The primary antibodies and dilutions used in IHC were S1PR1 (1:300; cat^#^ MABC94, Millipore), CD31 (1:50; cat^#^ AB28364, Abcam), Iba-1 (1:500; cat^#^ 019-19741, Wako), pTau (AT8) (1:500; cat^#^ MN1020, Thermo Scientific), and NeuN (1:500; cat^#^ ABN78, Millipore). Cy™3-conjugated AffiniPure Goat anti-rat IgG (1:50; cat^#^ 112165167; Jackson ImmunoReaserch) was used to stain serum IgG extravasation.

For immunofluorescence, 10 µm sections of frozen rat brain were treated with acetone and blocked with 5% normal goat serum. Primary antibodies were incubated for 24 h at 4°C. Sections were incubated for 90 min at 25°C with secondary antibodies conjugated with FITC or Cy-3 (cat^#^ A11029, A11034, A11032, and A11037; Invitrogen). 4’,6-diamidino-2-phenylindole (DAPI) (cat^#^ D3571, Molecular Probes) was used to label cell nuclei. Immunohistochemistry (IHC) negative controls were incubated without the primary antibody or with normal (non-immune) IgGs and no specific immunolabelling was detected.

All IHC slides were viewed on an Olympus BX-51 bright field and fluorescence microscope (Olympus America Inc.). Dual or triple immunofluorescence slides were also imaged with Nikon ECLIPSE TiS Inverted Microscope capable of 3D (motorized XY stage and Z focus) Image Stitching for both Bright-field and Single Wave-length/filter cube Epi-Fluor and its software (Nikon Instruments Inc.). Dual immunofluorescence slides were also imaged confocally to verify co-labelling (Zeiss LSM 800, Carl Zeiss Microimaging).

### Quantification of Iba-1 fluorescence density and colocalization of S1PR_1_ with CD31.

The staining density of Iba-1 immunofluorescence was measured to quantify the activation of microglia as described before [40]. Rats were euthanized and perfused at 4 and 9 weeks after UCAO/JPD onset. Eight brain sections from each animal with an interval of 100 μm covered a span of 800 μm in the region of the Bregma (-0.80 to -3.8 mm). Microglia/macrophages labeled with Iba-1 were measured in images captured from ischemic hemispheres with a low power objective lens (10X) by using ImageJ (National Institutes of Health). Indicators of animal identity on the slides were blinded to the investigator. The intensity of Iba-1 fluorescence was calculated as the mean of the intensity obtained from imaged sections.

For analysis and quantification of S1PR_1_ expressed in endothelial cells (CD31), 8 brain sections from each animal were used as described above. Three areas from the dorsal cortex, WMs, and hippocampus were captured with a 20X objective lens. Automatic and quantitative measurement of colocalization of S1PR_1_ and CD31 was performed using the colocalization plugin, Coloc 2, in Fiji-ImageJ (http://fiji.sc/Coloc2), as we did before [40]. Briefly, 2-color channel images were converted into 8-bit grey images. After background subtraction, colocalization analysis was run in Coloc 2. A corresponding two-dimensional intensity histogram was also presented. Among multiple results, we chose to report Li’s Intensity correlation quotient (ICQ), which provides an overall index of colocalization. The ICQ values are distributed between -0.5 and +0.5 by subtracting 0.5 from this ratio. Random staining: ICQ~0; Segregated staining: 0 > ICQ -0.5; colocalized staining: 0 < ICQ ≤ +0.5 [46].

### DAB staining and quantification for S1PR_1_ in human brain tissue

Chromogen 3,3′-Diaminobenzidine (DAB) IHC was performed on the brain sections of SVD patients for S1PR_1_ expression and quantification. DAB is commonly employed as a staining agent in histochemical and immunohistochemical procedures performed for clinical and research purposes [47].

Human brain specimens from patients that underwent routine autopsy were fixed in 10% formalin for neuropathological examination. Brain blocks obtained at brain cutting were prepared for paraffin embedding. Blocks were microtome sectioned and 4 μm sections were mounted on slides. Sections for DAB staining were defatted in two changes of xylenes for 5 minutes each. Antigen retrieval was performed using the hot citrate buffer method. Briefly, slides were rehydrated through a graded alcohol series to a final rinse in tap water before being incubated in a citrate buffer (10 mM sodium citrate, 0.05% Tween-20, pH 6.0) heated to 100°C for 30 minutes. Slides were removed from the incubator and the citrate buffer was allowed to cool to room temperature after which the slides were rinsed in PBS-T for 5 min. Sections were blocked with 5% normal goat serum for 30 min. Then, sections were incubated with primary mouse anti-S1PR_1_ antibody (1:300; cat^#^ MABC94, Millipore) for 24 hours at 4°C. Development of IHC was carried out using Dako Liquid DAB + Substrate Chromogen System (Agilent Technologies) according to the manufacturer’s recommendations. Cresyl violet acetate counterstain was performed for nuclear labelling. Sections were washed in tap water and dehydrated in graded ethanol solutions (30 sec in 70%, 30 sec in 90%, and 3 min in 100% ethanol) followed by 3 min in xylene. DAB staining without the primary antibody for S1PR_1_ was performed as negative control.

S1PR_1_ expression in WMs and GMs was quantified using the 'Colour Deconvolution' plug-in in ImageJ (https://imagej.net/Colour_Deconvolution). Images of pathological and healthy brain sections were captured with a 40X power objective lens. For each case, six images were analysed. The percentage of area stained with DAB-positive for S1PR_1_ was calculated as a mean obtained from the imaged sections.

**Figure 1.**
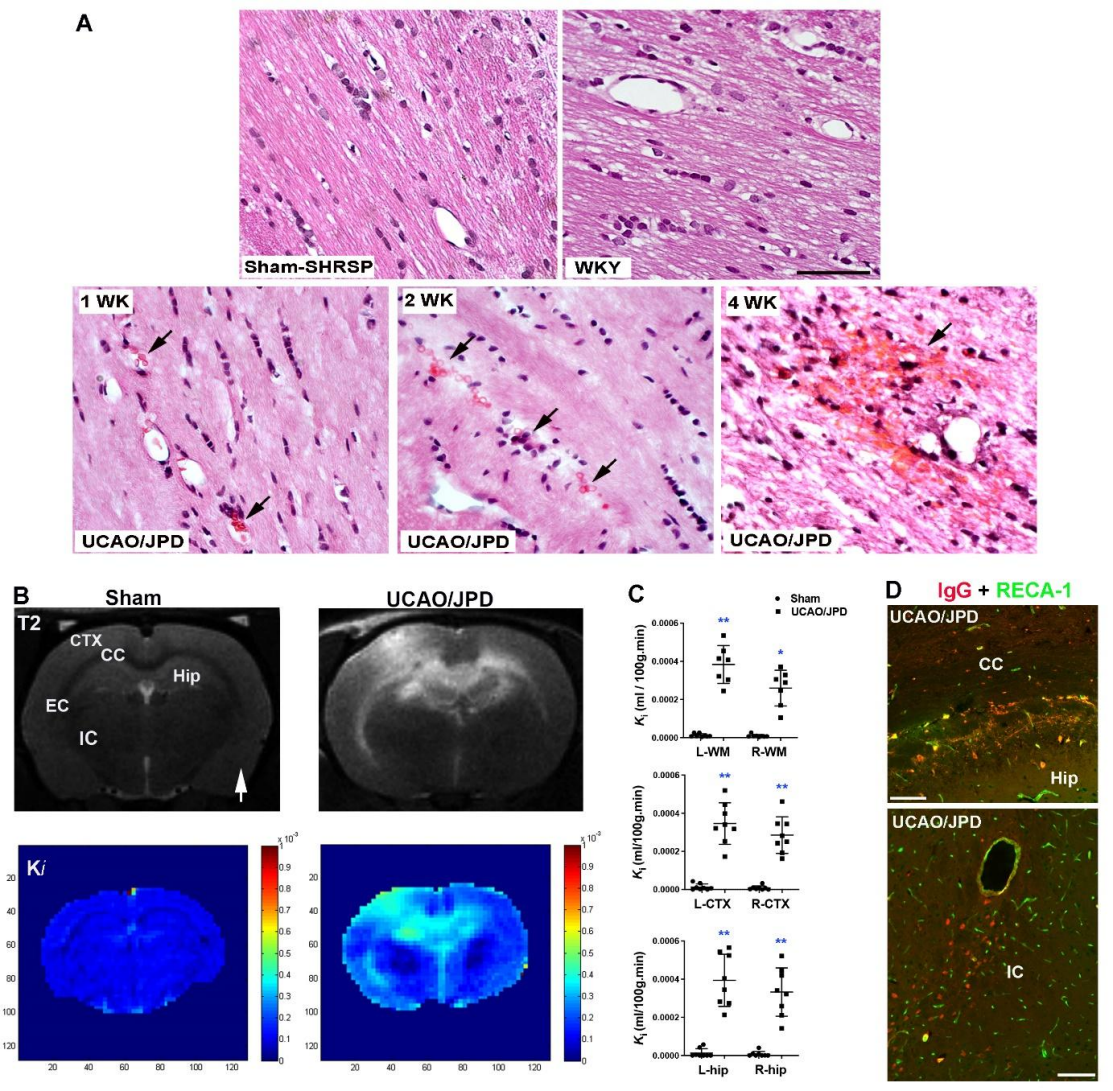
**Microbleeds and BBB disruption in white and grey matters in SHRSP rat brains 4 weeks after UCAO/JPD onset**. (**A**) H&E staining shows the development of microbleeds in CC from 1 - 4 weeks after UCAO/JPD onset, compared with sham SHRSP and WKY (control for the SHRSP) rats. Arrows indicate the extravascular red cells. Scale bar = 50 μm. (**B**) White and grey matter lesions and BBB leakage monitored by MRI in rats at 4 weeks after UCAO/JPD. Upper panels: Anatomical T2-weighted (T2) images. The arrow indicates the right hemispheres. CTX: dorsal cortex, CC: corpus callosum, EC: external capsule, IC: internal capsule, Hip: hippocampus. Bottom panels: parametric image *K*_i_ map represents BBB transfer rate for BBB permeability from the same rat brains in upper panels. Color-coded permeability coefficient maps reconstructed from DCE-MRI data demonstrate the regions of high (red) and low (blue) permeability. (**C**) Quantification of BBB transfer rate (*K*_i_) in white matter (WM), dorsal cortex, and hippocampus, in left (L) and right (R) hemispheres. WM: **p < 0. 01 vs. Sham L-WM; *p < 0.05 vs. Sham R-WM. CTX: **p < 0. 01 vs. Sham L- and R-CTX. Hip: **p < 0. 01 vs. Sham L- and R-hip. n=6 in Sham group, n=8 in UCAO/JPD group. (**D**) BBB leakage by double-immunostaining for serum IgG (red) extravasation and RECA-1 (green) in corpus callosum, hippocampus, and internal capsule in UCAO/JPD SHRSPs. RECA-1 is a cellular marker for vascular endothelial cells. Scale bars=50 μm.

### Western blots

Our MRI images demonstrated that WM lesions (seen in the corpus callosum, external capsule, and internal capsule) and grey matter (GM) lesions (seen in the cortex and hippocampus) are related to BBB leakage development in UCAO/JPD SHRSPs. Western blots were performed to determine protein levels in the affected WM and GM. Frozen coronal sections from another set of the 3 designed groups were cut at 400 micrometer thicknesses and micropunched biopsies were collected from both brain hemispheres in the dorsal cortex (DCTX), WMs (including the external capsule (EC), corpus callosum (CC), and internal capsule (IC), and hippocampus (HIP)) at -25 °C [36]. Proteins were extracted in RIPA buffer and 50 µg of total proteins were separated on 4% to 20% gradient gels (Bio-Rad). The proteins were transferred to polyvinylidene fluoride (PVDF) membranes. The membranes were then incubated with primary antibodies: S1PR_1_ (1:1000, cat^#^ MABC94, Millipore), S1PR_2_ (1:500; cat^#^ PA5-23208, Invitrogen), pAkt (1:1000; cat^#^ 4058S, Cell Signaling), total Akt (1:1000; cat^#^ 9272S, Cell Signaling), pTau (AT8, 1:500; cat^#^ MN1020, Thermo-Fisher Scientific), total Tau (Tau5, 1:1000; cat^#^ AHB0042, Invitrogen), pErk1/2 (1:1000; cat^#^ 9101S, Cell Signaling), total Erk1/2 (1:1000; cat^#^ 9102S, Cell Signaling), claudin-5 (1:500; cat^#^ 35-2500, Invitrogen), occludin (1:500; cat^#^ 71-1500, Invitrogen), and ZO-1 (1:500; cat^#^ 61-7300, Invitrogen). The membranes were incubated with their respective secondary antibodies, and blots were developed using the ECL Western Blotting Substrate (cat^#^ 32106, Thermo Fisher Scientific). Protein bands were visualized on X-ray film. Semiquantitation of target protein intensities was done with the use of ImageJ (NIH) and actin (1:7500; cat^#^ A5060, Sigma-Aldrich) immunoblots on the same PVDF membranes were used to normalize protein loading and transfer. The results are reported as normalized band intensity with β-actin (ratio of target protein band intensity to β-actin band intensity) for quantifying relative level of protein expression.

### Statistics

The Shapiro-Wilk test and Kolmogorov-Smirnov test were used first to determine whether the data deviated from Gaussian distributions. Unpaired t-test or non-parametric Mann-Whitney test (N<6, or non-Gaussian distribution data) were used for comparison between the two treatments. One-way ANOVA with post-hoc by Tukey’s post-hoc test (N≥6) or non-parametric Kruskal-Wallis with Dunn’s test (non-Gaussian distribution data) were used for comparison between multiple treatments. In all statistical tests, differences were considered significant when p < 0.05. Data are presented as means ± SD. The n indicates the number of individual animals used in each group. Statistical analyses were performed using Prism, version 9.5.0 (GraphPad Software Incorporated).

## RESULTS

### UCAO/JPD induced chronic endothelial injury and microbleeds, which are spatially associated with BBB leakage in SHRSPs

To examine whether chronic hypoxia induces endothelial dysfunction, we first detected erythrocyte accumulation in capillaries and vessel wall microbleeds, using H&E staining. SHRSP rats with UCAO/JPD were sacrificed at 1, 2, and 4 weeks after UCAO/JPD onset. Age-matched (16 weeks old) normotensive Wistar Kyoto (WKY) rats and sham SHRSP rats were employed as controls (Fig. 1A). Erythrocyte accumulation was seen in capillaries of lesioned WM at week 1, while increasing microbleeds were seen in lesioned WMs from 2 to 4 weeks after UCAO/JPD onset. No erythrocyte accumulation and microbleeds were detected in sham SHRSP rats and normotensive WKY rats. At week 4, compared with sham rats, UCAO/JPD rats demonstrated significantly increased BBB leakages (*K*_i_: blood-to-tissue transfer) in the lesioned dorsal cortex, WMs (corpus callosum, external capsule, and internal capsule), and hippocampus in both hemispheres (Fig. 1B and C) by DCE-MRI. The MRI observations corresponded with histology of serum IgG extravasation in lesioned WM and GM (Fig. 1D). The BBB leakages spatially correlated with the lesions (hyperintensities) in WMs, GMs, and hippocampus detected by MRI T2-weighted images in the same rat brain. These results suggested that UCAO/JPD chronically induced capillary injury and endothelial dysfunction, leading to BBB leakage and eventual brain damage in SHRSP rats.

### UCAO/JPD induced progressive neuroinflammation and phospho-Tau protein (pTau) accumulation

**Figure 2.**
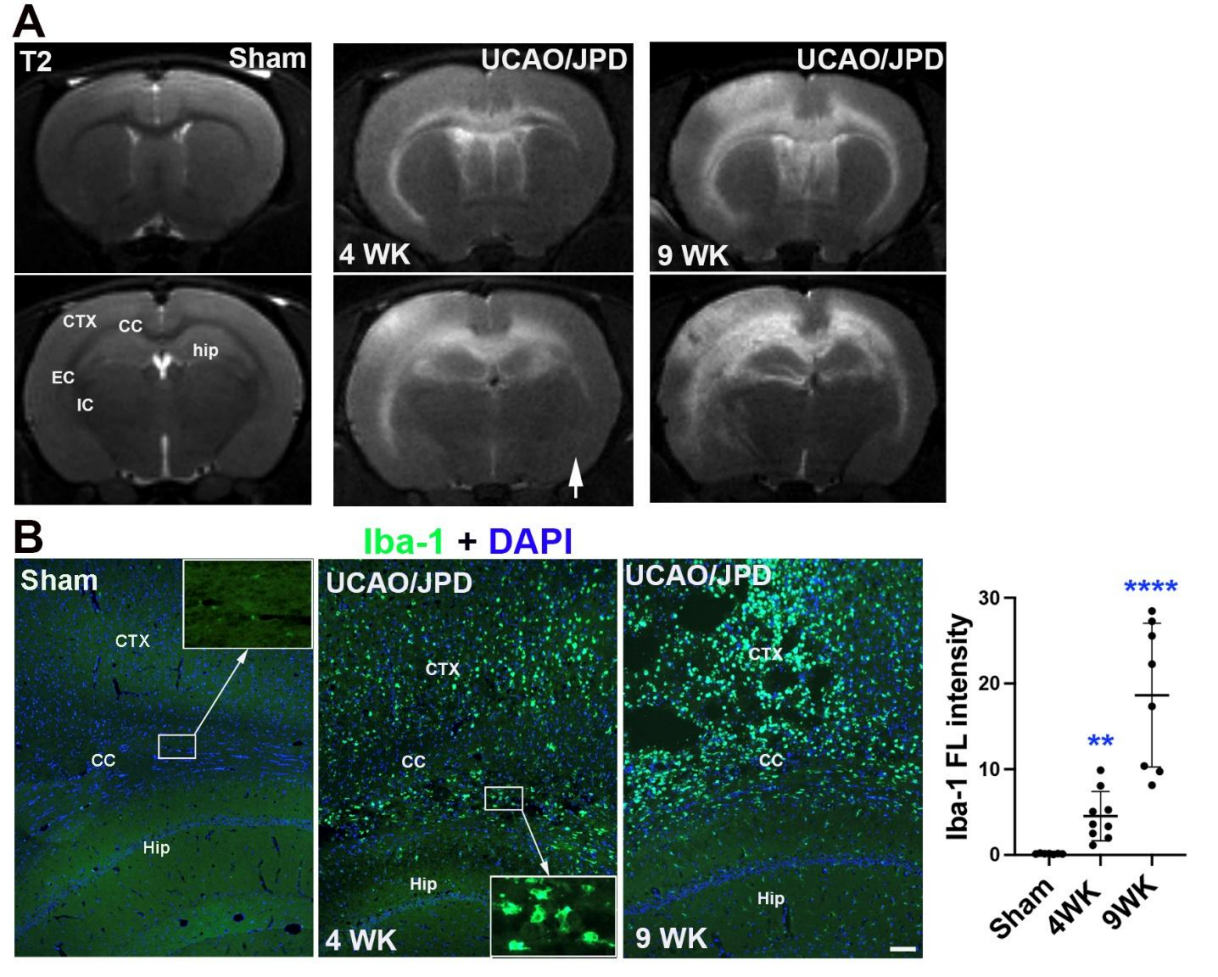
**Progressive white and grey matter lesions and inflammation development in rat brains from 4-9 weeks after UCAO/JPD**. (**A**) MRI T2-weighted images monitored the increased extent of lesions in the white and grey matters in a rat brain from 4-9 weeks compared with an age-matched sham rat. The arrow indicates the right hemisphere. (**B**) Representative photomicrographs show immunostaining of Iba-1, a cellular marker of active microglia, in rat brains subjected to 4 or 9 weeks UCAO/JPD compared with sham. Inserts present higher magnification images. DAPI was used to show nuclei. Scale bar = 10 μm. Graph shows the quantification of Iba-1 fluorescent (FL) intensity. **p < 0.01 vs. sham, ****p < 0.0001 vs. Sham and 4WK groups. n=6 in sham, n=9 in 4WK, and n=8 in 9WK groups.

We previously reported that when UCAO/JPD was extended to 9 weeks, the rats developed significantly larger and more extensive WM lesions than those observed at 4 weeks [34]. These progressive lesions were also seen in the cortex and hippocampus, shown as hyperintensities in T2-weighted images (Fig. 2A). The progressive lesions in GMs were also reported in human SVD [48]. Using an antibody for Iba-1, a cellular marker for active microglia/macrophages, we examined the neuroinflammatory response to UCAO/JPD induced chronic hypoxia. In line with the extent of brain injuries detected by MRI T2-weighted imaging, immune-histochemical staining of Iba-1 demonstrated a progressive increase of active microglia/macrophages in the lesioned WMs and GMs from week 4 to week 9 compared with sham rats (Fig. 2B). Iba-1 is specifically expressed in microglia/macrophages and is upregulated during activation of these cells in injured brains [49, 50]. Quantifying the density of Iba-1 immunofluorescence proportionally reflects both Iba-1^+^ cell number and level of Iba-1 protein in microglia/macrophages [50]. These active microglia/macrophages and reactive astrocytosis express inflammatory cytokines and factors, such as matrix metalloproteinases (MMP)-3, MMP-9, and TNF-α in lesioned WMs in the rat model [35, 51]. Compared with sham, UCAO/JPD induced significantly higher activation of microglia/macrophages in WMs, cortex, and hippocampus over 4 to 9 weeks, while significantly larger and more extensive inflammation was developed at 9 weeks compared with those observed at 4 weeks.

**Figure 3.**
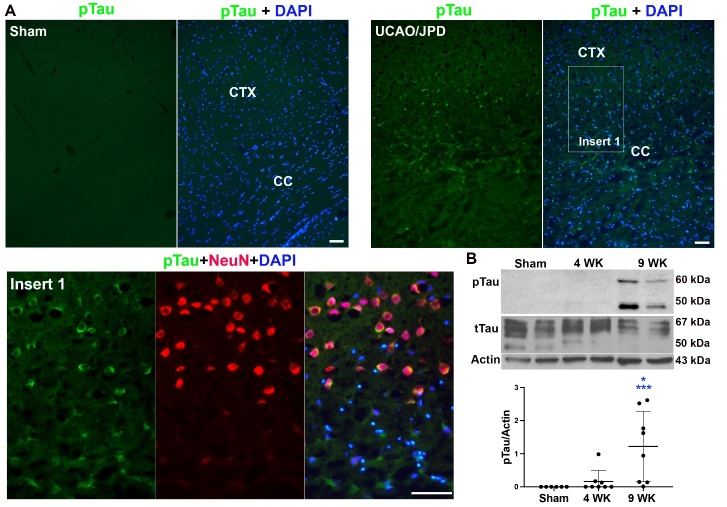
**Progressive AT8 (pTau Ser202/Thr205) accumulation in rat brains subjected to UCAO/JPD**. (**A**) Photomicrographs represent expression of pTau in sham rats and SHRSP rats 9 weeks after UCAO/JPD. Insert 1 shows accumulation of AT8 in neurons of the cortex area adjacent to the lesioned CC. Scale bars = 50 μm. (**B**) Western blot analysis of protein levels for AT8 in rat cortices from 4-9 weeks after UCAO/JPD. *p < 0.05 vs. 4WK, ***p < 0.001 vs. Sham. n=6 in sham, n=8 in 4WK and 9WK groups.

Brains of patients with vascular dementia and vascular injury demonstrated accumulation of phosphorylated tau protein [52, 53]. Using an early tangle marker, AT8, we examined if there was an accumulation of phosphorylated tau (pTau) protein in rat brains subjected to UCAO/JPD. Histologically, positive staining of pTau was seen in lesioned cortex and corpus callosum at 9 weeks after UCAO/JPD onset, while double-immunostaining demonstrated pTau accumulation in neurons located within layers V and VI of the cortical areas, where the neurons are suggested for maintenance and control of working memory [54] (Fig. 3A). Further Western blot analysis showed that, compared with sham rats, increased pTau protein levels were seen at 4 weeks and significantly elevated at 9 weeks in UCAO/JPD groups (Fig. 3B). The accumulation of pTau that occurred over 4 to 9 weeks after UCAO/JPD onset was spatiotemporally consistent with the increased microglia/ macrophage activation in the lesioned rat brains.

Besides WM lesion, cognitive impairment, BBB disruption, and inflammation [35-38], the results above indicate that our UCAO/JPD SHRSP rat model developed some major neuropathological hallmarks of human SVD [55, 56], including endothelial injury, microbleeds, progressive activation of microglia/ macrophages, and accumulation of neuronal pTau in response to chronic hypoxic hypoperfusion.

### UCAO/JPD induced progressive endothelial S1PR_1_ disruption in SHRSPs and decreased expression of S1PR_1_ in cerebral vessels of human SVD

**Figure 4.**
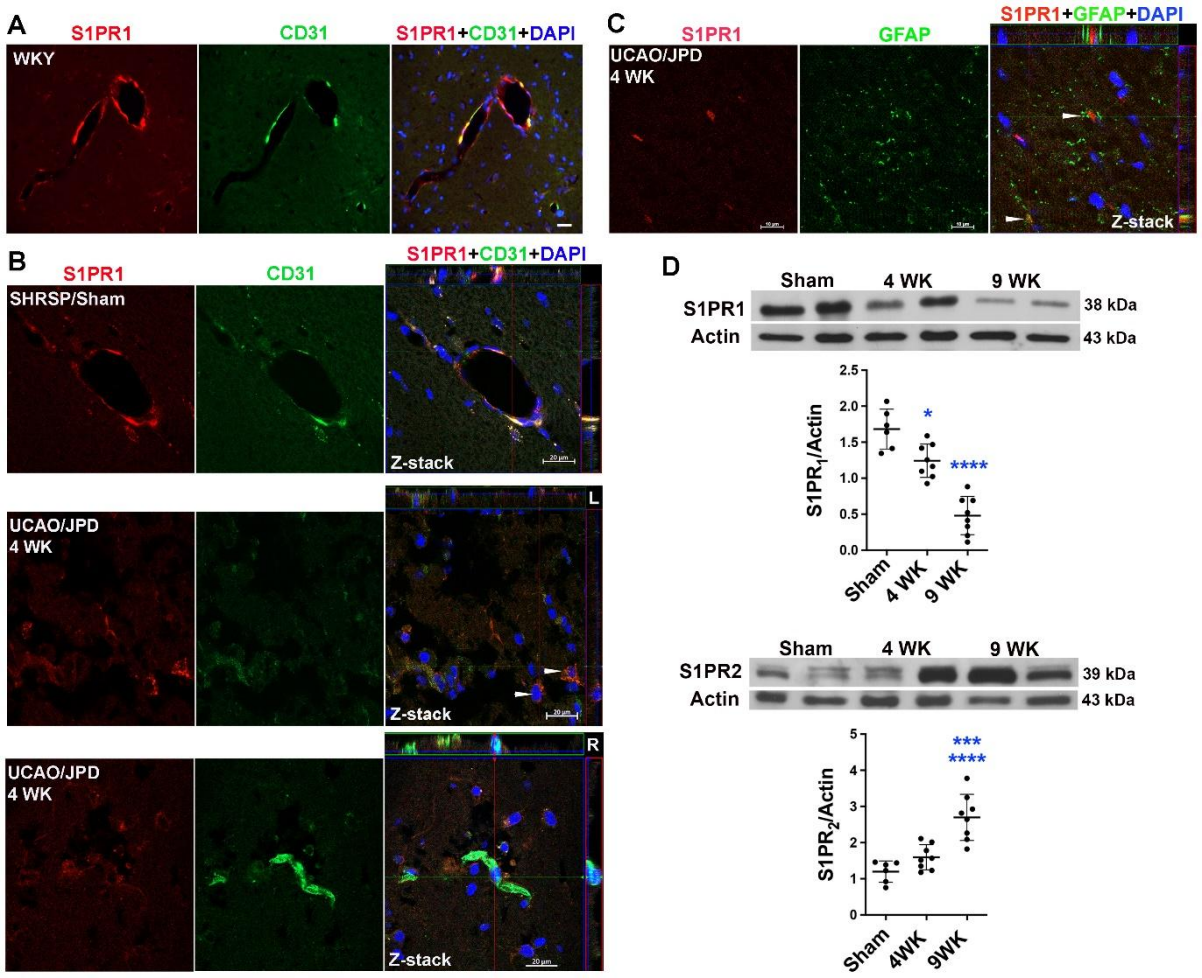
**Progressive loss of endothelial S1PR_1_ in rat brains subjected to UCAO/JPD**. (**A**) Photomicrographs represent double immunostaining of S1PR_1_ with CD31 (a cellular marker of endothelial cells) in white matter of a WKY rat. DAPI was used to show nuclei. Scale bar = 20 μm. (**B**) Z-stack confocal photomicrographs represent double immunostaining of S1PR_1_ expression in endothelial cells in the white matters (corpus callosum) of sham rat and SHRSP rats 4 weeks after UCAO/JPD. L: left hemisphere, R: right hemisphere. (**C**) Z-stack confocal photomicrograph represents double immunostaining of S1PR_1_ expression in GFAP-positive astrocytes (arrows) in the lesioned white matter (corpus callosum). (**D**) Western blot analysis of protein levels for S1PR_1_ and S1PR_2_ in rat brains subjected to UCAO/JPD for 4 or 9 weeks. S1PR_1_: *p < 0.05 vs. sham, ****p < 0.0001 vs. Sham and 4WK. S1PR_2_: ****p < 0.0001 vs. Sham, ***p < 0.001 vs. 4WK. n=6 in Sham, n=8 in 4 WK and 9WK groups.

Endothelial injury and inflammation lead to degradation of S1PR_1_ [25, 57]. To gain insight into the impact of chronic hypoxic hypoperfusion on cerebral endothelial S1PR_1_, we first examined the expression of S1PR_1_ in vascular endothelial cells (ECs) in the WMs of WKY rats. Double IHC and confocal microscopy demonstrated the co-localization of S1PR_1_ with ECs stained with the antibody for CD31, a cellular marker of ECs, in WYK rat (Fig. 4A). Z-stack confocal images showed S1PR_1_ expression in ECs and that ECs are the major source of S1PR_1_ in rat brains (Fig. 4B). However, weaker expression of endothelial S1PR_1_ was seen in SHRSPs compared with expression in WKY rats. At week 4 after UCAO/JPD, a loss of S1PR_1_ from ECs was observed in the WM lesion areas by using confocal Z-stack analysis (Fig. 4B), while an increase of S1PR_1_ expression was seen in the surrounding reactive astrocytes (Fig. 4C). Accordingly, Western blot analyses revealed significantly diminished S1PR_1_ levels in UCAO/JPD rats compared with those of sham (Fig. 4D). When we extended our study to rats 9 weeks after UCAO/JPD we observed a significantly greater decrease in S1PR_1_ compared with those observed at 4 weeks. Since S1PR_2_ plays pivotal roles in CNS autoimmunity, cell differentiation, and enhancement of BBB permeability and leukocyte entry [26, 30, 31], we also measured protein levels of S1PR_2_. Significantly increased expression of S1PR_2_ was observed at 9 weeks after UCAO/JPD onset (Fig. 4C), making it likely that this UCAO/JPD-induced change in S1PR_2_ occurs later than for S1PR_1_, which indicates roles for S1PR_2_ in inflammation and astrogliosis.

**Figure 5.**
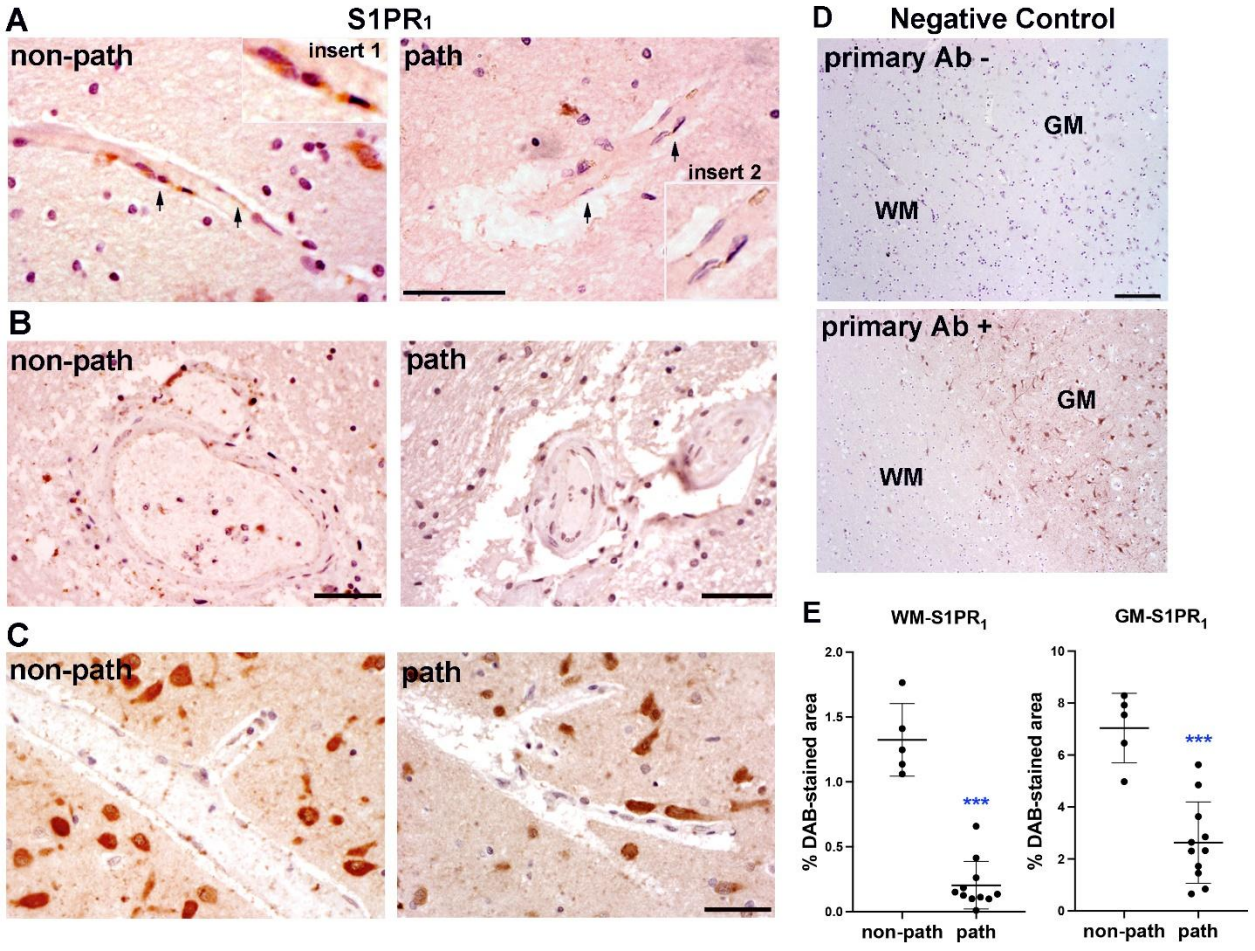
**DAB immunostaining for S1PR_1_ expression in autopsy brain specimens of SVD patients**. (**A**) S1PR_1_ expression in capillary endothelial cells in pathological WM and non-pathological WM. Arrows indicate the endothelial cells that expressed S1PR_1_. Inserts 1 and 2: higher magnification of the endothelial cells with S1PR_1_. (**B**) S1PR_1_ expression in endothelial cells of arterioles and veins in pathological WM and non-pathological WM. (**C**) S1PR_1_ expression in neurons and capillary endothelial cells in pathological GM and non-pathological GM. (**D**) DAB immunostaining with or without primary antibody for S1PR_1_. primary AB -: no primary antibody for S1PR_1_ was used; primary AB +: primary antibody for S1PR_1_ was used. Scale bars = 50 μm. (**E**) Graph demonstrates quantification of the percentages of DAB-stained areas in WMs and GMs, respectively. ***p < 0.001 vs. non-path WM; ***p < 0.0001 vs. non-path GM. n=5 in non-path groups, n=11 in path groups. path: brain areas from the patients with particular emphasis on the sites of SVD/VCID pathology in the WM and GM. non-path: healthy brain areas from these cases were used as non-pathological controls.

To gain insight into the morphological and molecular relevance of S1PR_1_ changes in human SVD, we next examined the expression of S1PR_1_ in the autopsy brain specimens of SVD patients using DAB IHC staining. In the non-pathological brain tissue (control), positive S1PR_1_ signal was observed in vascular ECs in WM and GM (Fig. 5A, B, and C, left panels). Within WM and GM lesion areas, capillary ECs showed degradation of S1PR_1_ (Fig. 5A and C, right panels), which was also seen in the arterioles and neurons (Fig. 5B and C, right panels). DAB staining with and without primary antibody for S1PR_1_ was performed as a negative control (Fig. 5D). We used the percentage of DAB-positive areas to quantify the expression of S1PR_1_ in human brains as previously described [53, 58]. Compared with the non-pathological brain tissues, significant decreases of S1PR_1_ expression were detected in the lesioned WM and GM areas (Fig. 5E).

These animal and human data suggest that chronic hypoxic hypoperfusion triggers disruption of S1P-S1PR_1_ signalling, leading to endothelial injury, BBB dysfunction in SVD, and the potential of the S1P-S1PR_1_ signalling pathway as a protector of vascular function to prevent BBB leakage and the progressive development of SVD.

### SEW2871 reduced lesions in WM and GM, and improved cerebral blood flow (CBF) and protected endothelial S1PR_1_ from degradation in UCAO/JPD SHRSPs

The tetraaromatic compound SEW2871 is a selective agonist of S1PR_1_ [59, 60]. Seven-day treatment with SEW2871 (0.5 mg/kg/day IP) improved cognitive function in the rat model of AD [61] and enhanced neurogenesis in the rat model of TBI [39]. Due to the development of WM lesions from 21 to 28 days in our rat model [51], we chose to use the same IP dose every other day for 28 days and treated the rats with either SEW2871 or vehicle.

**Figure 6.**
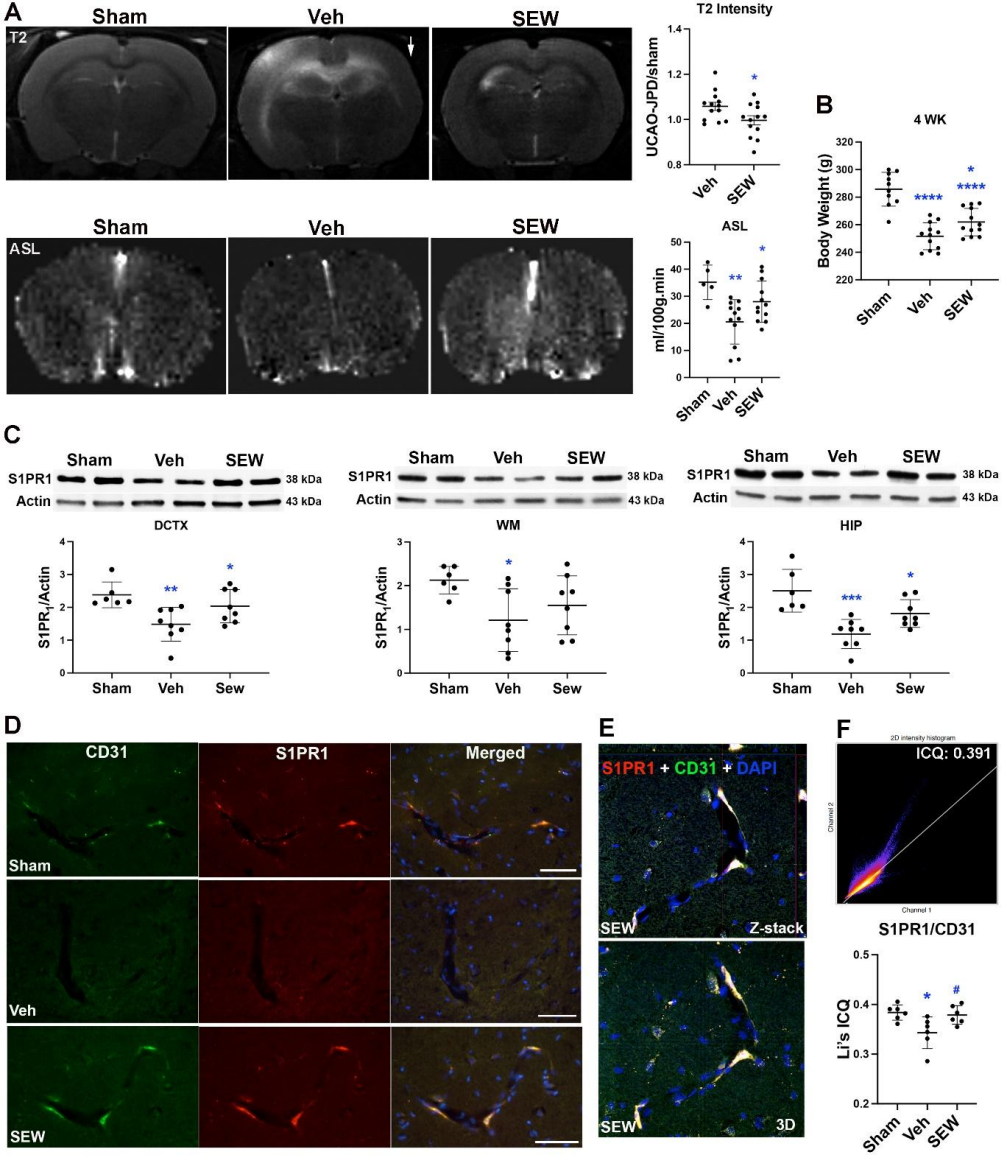
**SEW2871 preserved the endothelial S1PR_1_ and attenuated lesions in WM and GM at 4 weeks following UCAO/JPD**. (**A**) Representative MRI T2-weighted images and ASL maps. The arrow indicates the right hemisphere. Graphs demonstrate quantification of T2-weighted intensity and CBF in rat brains of sham-, vehicle-, and SEW2871-treated groups. *p < 0.05 vs. Veh, **p < 0.01 vs. Sham. n=6 in sham group, n=12-13 in vehicle and SEW2871 groups. (**B**) Body weight measurement for rats from rats in sham, vehicle-treated, and SEW2871-treated groups, respectively. n=10 in Sham group, n=12 in Veh and SEW2871 groups. (**C**) Western blot analysis for protein level for S1PR_1_ in WMs, dorsal cortex, and hippocampus of sham-, vehicle-, and SEW2871-treated groups. Graphs show that SEW2871 enhanced the protein levels of S1PR_1_. DCTX: *p < 0.05 vs. Veh, **p < 0.01 vs. Sham. WM: *p < 0.05 vs. Sham. HIP: *p < 0.05 vs. Veh, ***p < 0.001 vs. Sham. n=6 in sham, n=8 in vehicle and SEW2871 groups. (**D**) Representative IHC images show the expression of S1PR_1_ in endothelial cells (CD31) in white matter (internal capsule) of sham rat, vehicle, and SEW2871 treated rats 4 weeks after UCAO/JPD. Scale bar = 50 μm. (**E**) Z-stack and 3D confocal images show colocalizations of S1PR_1_ with CD31-positive ECs in rat WM treated with SEW2871. (**F**) Analysis and quantification for colocalization of S1PR_1_ and CD31 in the endothelial cells with Fiji-ImageJ. Representative two-dimensional histogram and scatterplot visualize the correlation of the pixel intensities over all pixels and voxels in the images with different Li’s ICQ values. Statistical bar figures demonstrate the quantification of Li’s ICQ values for colocalization of S1PR_1_ with CD31 in sham, vehicle-, and SEW2871-treated white matter. **p < 0.01 vs. Veh, # p < 0.05 vs. Veh. n = 6 in each group.

To examine the protective role of SEW2871 on UCAO/JPD induced brain damage in SHRSP rats, we first used MRI imaging to measure the changes of brain injury and CBF in the SHRSP rats at 4 weeks after UCAO/JPD. Hyperintensity lesions in WM and GM were detected at day 28 using T2-weighted MRI scans. Twelve T2-weighted images were generated for each rat brain. Intensity values of each T2-weighted image were measured by ImageJ and an average intensity was generated from the 12 T2-weighted images. To correct any potential asymmetry of the intensity between right and left brain regions due to the inherent radiofrequency inhomogeneity of the surface coil used to obtain the images [62], the intensity values in vehicle and SEW2871 groups were presented as intensity ratios to sham group. Significantly lower hyperintensity lesions in WM and GM was seen in SEW2871-treated rats compared with the vehicle group (Fig. 6A, top panels). The MRI arterial spin labelling (ASL) map for cerebral blood flow (CBF, mL/100g.min) was calculated using Perfusion Processing macro in ParaVision 5.1. As we reported before, UCAO/JPD significantly reduced CBF in SHRSP rats compared with those in sham group, while treatment with SEW2871 significantly increased CBF compared with the vehicle group (Fig. 6A, bottom panels). In addition to the neuroprotective role, our data also demonstrated that treatment with SEW2871 significantly attenuated UCAO/JPD induced body weight loss in the SHRSP rats (Fig. 6B).

Next, using Western blot, we analyzed the changes of S1PR_1_ protein levels in WMs and GMs of the rat brains treated with vehicle or SEW2871. At 4 weeks after UCAO/JPD onset, significant decreases of S1PR_1_ protein levels were detected in the dorsal cortex (DCTX), WM (including corpus callosum, external capsule, and internal capsule), and hippocampus (HIP) (Fig. 6C), where MRI T2-weighted images showed hyperintensity lesions in the vehicle brain (Fig. 6A). Treatment with SEW2871 reversed the reduction of S1PR_1_ protein levels in these lesioned areas, while significantly higher levels were detected in the dorsal cortex and hippocampus. Further double-immunohistochemical staining for S1PR_1_ and CD31 (marker of ECs) showed loss of endothelial S1PR_1_ induced by UCAO/JPD in the WM (internal capsule, IC), which was preserved by SEW2871 treatment (Fig. 6D and E). Quantification of colocalization of S1PR_1_ with CD31 demonstrated that SEW2871 treatment significantly ameliorated the loss of S1PR_1_ from microvascular ECs in WM (Fig. 6F).

These results indicate that the activation of S1PR_1_ by SEW2871 is a therapeutic target for vascular protection in UCAO/JPD model.

### SEW2871 preserved TJPs and reduced accumulation of pTau in the WMs and GMs in UCAO/JPD SHRSPs

**Figure 7.**
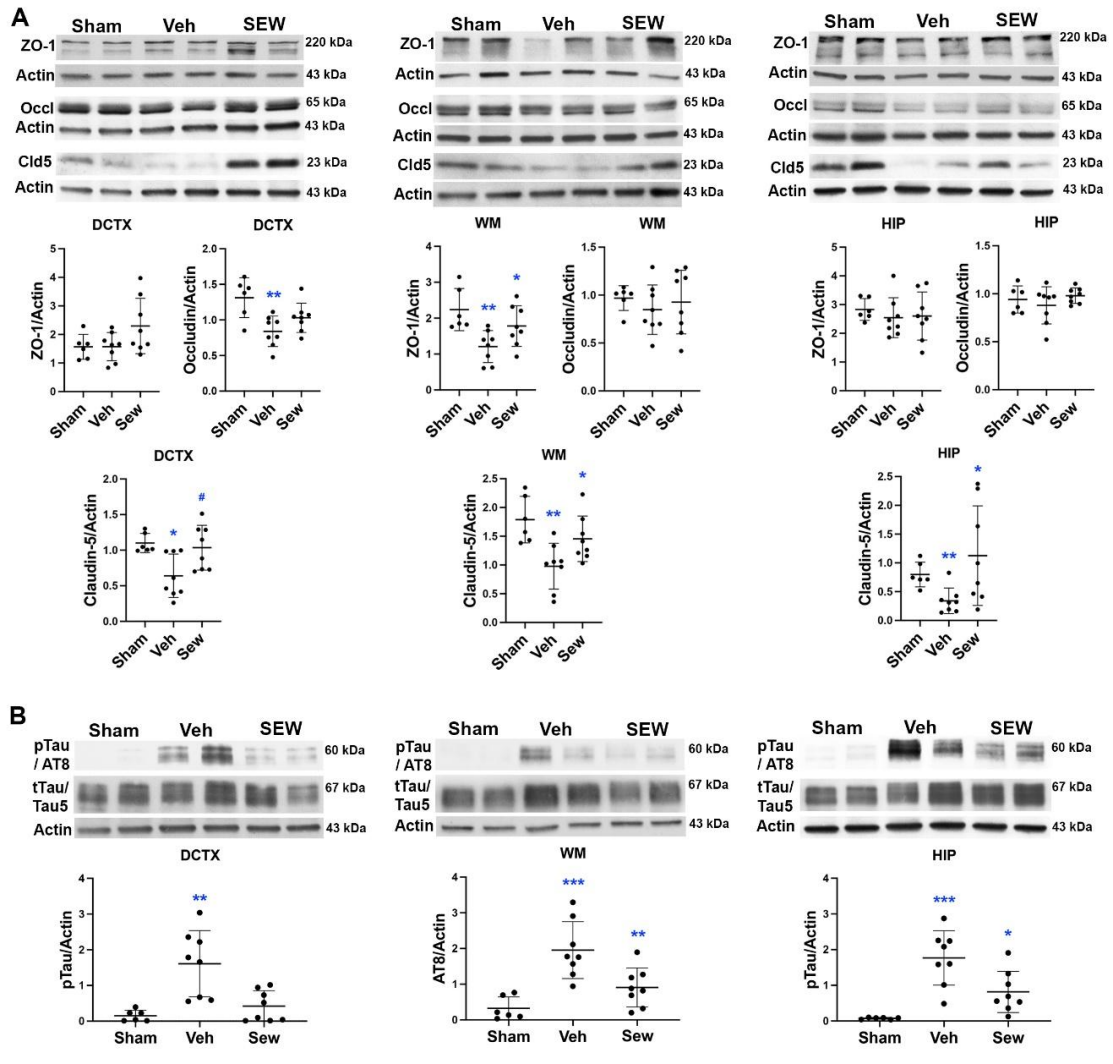
**SEW2871 preserved TJPs and attenuated pTau accumulation in WM and GM at 4 weeks following UCAO/JPD by Western blots**. (**A**) Blots and protein level measurement of TJPs, ZO-1, occludin (Occl), and claudin-5 (Cld5) in the WMs, dorsal cortex, and hippocampus from rats of sham-, vehicle-, and SEW2871-treated groups. DCTX: *p < 0.05 and **p < 0.01 vs. Sham, ^#^p <0.01 vs. Veh. WM: *p < 0.05 vs. Veh, **p < 0.01 vs. Sham. HIP: *p < 0.05 vs. Veh, **p < 0.01 vs. Sham. (**B**) Blots and protein level measurement of pTau (AT8) in WMs, dorsal cortex, and hippocampus from rats of sham-, vehicle-, and SEW2871-treated groups. DCTX: **p < 0.01 vs. Sham and SEW, WM: **p < 0.01 vs. Veh, ***p < 0.001 vs. Sham. HIP: *p < 0.05 vs. Veh, ***p < 0.001 vs. Sham. n=6 in sham, n=8 in vehicle and SEW2871 groups.

We previously reported histologically and biochemically significant disruption of TJPs, particularly claudin-5, in the vascular ECs within the lesioned WMs in SHRSPs at 4 weeks after UCAO/JPD. Using Western blot analysis, we measured the protein levels of TJPs, ZO-1, occludin, and claudin-5 in the dorsal cortex, WMs, and hippocampus to evaluate the effect of SEW2871 on the modulation of TJPs (Fig. 7A). Significantly decreased ZO-1 level in the vehicle group was detected in WM area compared with sham group, while treatment with SEW2871 significantly reversed the degradation of ZO-1 in WM. No significant changes of ZO-1 were observed in the dorsal cortex and hippocampus. UCAO/JPD significantly decreased occludin levels in dorsal cortex compared with sham, and treatment with SEW2871 showed protection of occludin from degradation. No significant changes in occludin levels were detected in WM and hippocampus. Consistent with our previous observation, UCAO/JPD significantly reduced claudin-5 in the lesioned dorsal cortex, WM, and hippocampus. Importantly, treatment with SEW2871 significantly enhanced claudin-5 protein levels in these areas. Previous studies have shown that claudin-5 is expressed only by vascular ECs in the brain [43, 63]. Our findings indicate that SEW2871 reversed the UCAO/JPD-induced claudin-5 degradation in ECs via activating endothelial S1PR_1_.

We next examined the impact of SEW2871 on pTau accumulation in the dorsal cortex, WMs, and hippocampus (Fig. 7B). Western blot showed remarkable accumulation of pTau in all three measured areas of rat brains subjected to UCAO/JPD at 4 weeks compared with sham brains. There was significant reduction of pTau levels in the dorsal cortex, WMs, and hippocampus with SEW2871 treatment compared with vehicle treatment. BBB dysfunction alters the inflammatory response and leads to microglial activity that is associated with pTau accumulation in the pathogenesis of SVD [64, 65]. Further study will be needed to test whether SEW2871 reduces pTau accumulation via the protection of BBB integrity in this model.

### SEW2871 attenuated the decrease of phospho-Akt (pAkt) and increase of phospho-Erk1/2 (pErk1/2), and it reduced S1PR_2_ protein levels in WMs and GMs in UCAO/JPD SHRSPs

**Figure 8.**
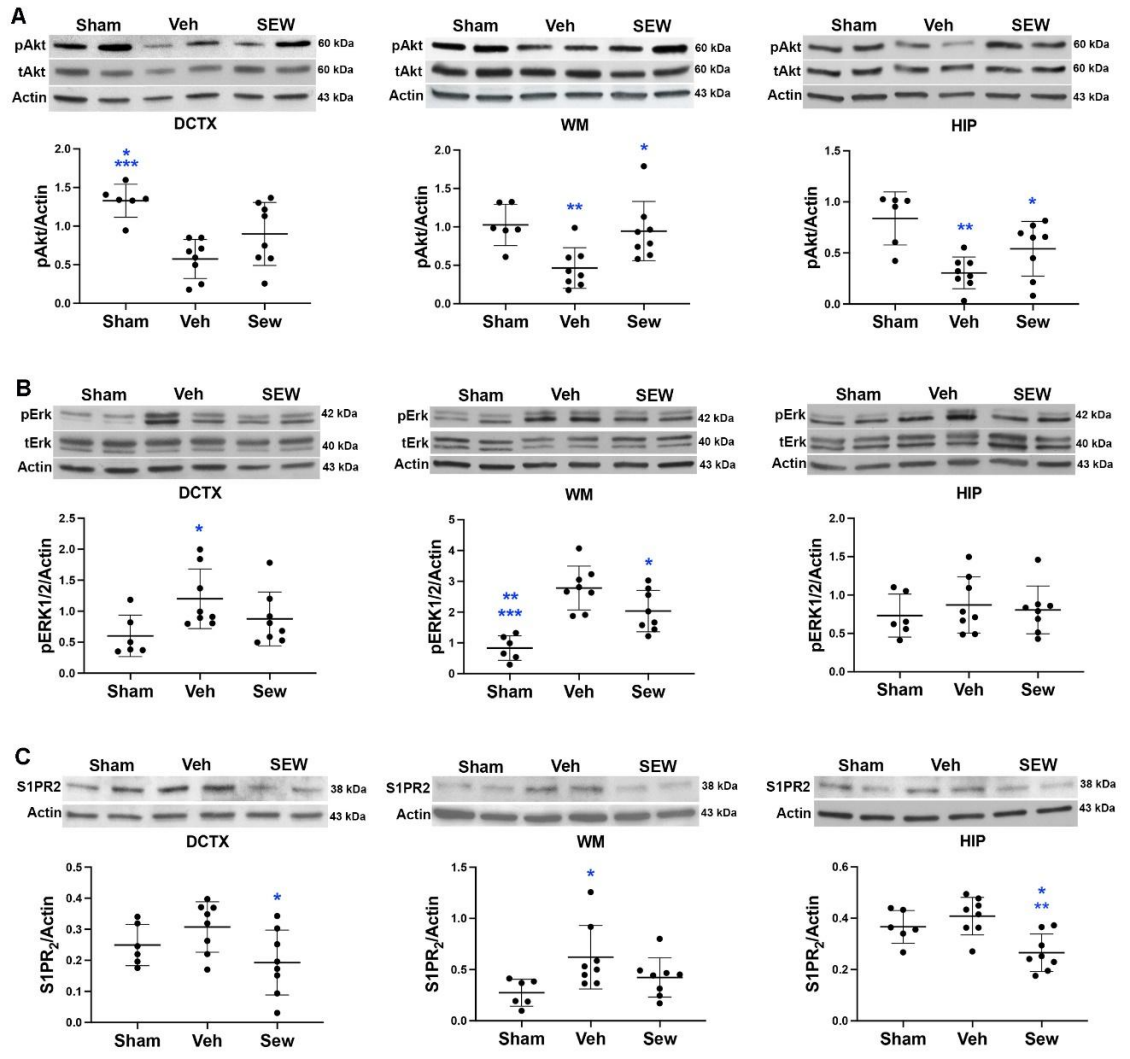
**SEW2871 promoted Akt phosphorylation and attenuated S1PR_2_/pErk1/2 in WM and GM at 4 weeks following UCAO/JPD by Western blots**. (**A**) Blots and protein level measurements of pAkt and tAkt in the WMs, dorsal cortex, and hippocampus from rats of sham-, vehicle-, and SEW2871-treated groups. DCTX: *p < 0.05 vs. SEW, ***p < 0.001 vs. Veh. WM: *p < 0.05 vs. Veh, **p < 0.01 vs. Sham. HIP: *p < 0.05 vs. Veh, **p < 0.01 vs. Sham. (**B**) Blots and protein level measurement of pErk1/2 and tErk1/2 in WMs, dorsal cortex, and hippocampus from rats of sham-, vehicle-, and SEW2871-treated groups. DCTX: *p < 0.05 vs. Veh and SEW. WM: *p < 0.05 vs. Veh, **p < 0.01 vs. SEW, ***p < 0.001 vs. Veh. (**C**) Blots and protein level measurement of S1PR_2_ in WMs, dorsal cortex, and hippocampus from rats of sham-, vehicle-, and SEW2871-treated groups. DCTX: *p < 0.01 vs. Veh. WM: *p < 0.05 vs. Sham. HIP: *p < 0.05 vs. Sham, ** p < 0.01 vs. Veh. n=6 in sham, n=8 in vehicle and SEW2871 groups.

Activation of S1PR_1_ couples with G_i/o_ and activates the PI3K/Akt pathway to prevent cellular apoptosis [66, 67], and phosphorylation of protein kinase B (Akt) could support cell survival, growth, migration, and angiogenesis. To test whether this cell survival pathway was involved in the protective role of SEW2871, we examined the change in pAkt protein levels in the dorsal cortex, WMs, and hippocampus subjected to UCAO/JPD with/without SEW2871 treatment (Fig. 8A). Western blot analysis demonstrated significantly decreased pAkt protein levels in the dorsal cortex, WM, and hippocampus subjected to UCAO/JPD compared with sham. Treatment of SEW2871 attenuated the reduction of pAkt induced by degradation. No UCAO/JPD, and a significant increase of pAkt was seen in WM and hippocampus treated with SEW2871 compared with those of the vehicle group. In addition to Akt, the downstream analysis has shown that S1PR_1_ activation also involves the phosphorylation of extracellular signal-regulated kinases 1/2 (Erk1/2) via the G_i/o_/Ras/Erk1/2 pathway to promote a pre-survival effect [68, 69]. We next examined the effects of UCAO/JPD and SEW2871 on pErk1/2 levels using Western blot (Fig. 8B). Unexpectedly, UCAO/JPD significantly increased pErk1/2 levels in the lesioned dorsal cortex and WMs at 4 weeks, compared with sham. SEW2871 treatment in rats reduced UCAO/JPD-elevated pErk1/2 levels in the three lesion areas, while significantly decreasing pErk1/2 levels were seen in the dorsal cortex and hippocampus subjected to SEW2871 compared with those subjected to vehicle. This finding indicates that UCAO/JPD induces elevation of Erk1/2 phosphorylation that is associated with brain damage, which would argue that S1PR_1_ activation triggers the pro-survival pathway via Ras/Erk1/2.

Previous report showed that S1PR_2_ antagonist alleviated oxidative stress-enhanced brain EC permeability by attenuating the Erk1/2 pathway [32]. In Fig. 4C, we demonstrated that UCAO/JPD significantly decreased S1PR_1_ levels at 4 weeks, while the observed S1PR_2_ levels were marginally greater in the UCAO/JPD brains, compared with sham, with the difference reaching statistical significance. S1PR_2_ enhances BBB permeability and leukocyte entry [26, 30, 31] via G_12/13_/Rho/Rock pathways, and inhibits S1PR_1_ [69-71]. Therefore, we examined if the treatment of SEW2871 affects S1PR_2_ expression at the same time in which differences in pErk1/2 and S1PR_1_ levels were apparent (Fig. 8C). Consistent with the finding in Fig. 4C, UCAO/JPD elevated the levels of S1PR_2_ in the lesioned dorsal cortex, WM, and hippocampus at 4 weeks compared with sham, without statistical difference. However, treatment with SEW2871 elicited a significant decrease in S1PR_2_ levels in the lesioned dorsal cortex, WM, and hippocampus. Our data suggested that S1PR_1_ activation by SEW2871 inhibited S1PR_2_ due to the antagonisms between S1PR_1_ and S1PR_2_, which is associated with the reduction of pErk1/2.

## DISCUSSION

In this study, we demonstrated that chronic hypoxic hypoperfusion disrupts endothelial S1PR_1_ in the cerebrovascular capillaries of a rat model of chronic hypoxic hypoperfusion with BBB dysfunction. These morphological and molecular changes of S1PR_1_ in rat brains were supported by a significant loss of S1PR_1_ examined histologically in autopsied brain specimens of SVD patients. Of pre-clinical significance, MRI analysis showed that treatment with the selective S1PR_1_ agonist, SEW2871, reduced lesions in the white matter (WM) and grey matter (GM), and improved CBF in SHRSPs. In agreement with the MRI findings, SEW2871 significantly attenuated the loss of capillary endothelial S1PR_1_, protected endothelial TJP, claudin-5, from degradation, accompanied by significantly decreased pTau accumulation in injured WM and GM, indicating reduction of BBB integrity disruption and neuronal lesion induced by UCAO/JPD. Further biochemical studies provided evidence that the neuroprotective role of SEW2871 is associated with facilitating Akt phosphorylation and inhibiting S1PR_2_ activation and Erk1/2 phosphorylation. To the best of our knowledge, the present findings are the first to describe endothelial S1PR_1_ signalling pathway as a potential cellular and molecular mechanism that links hypoxic hypoperfusion with BBB dysfunction in the progressive pathology of SVD, which could be reversible by selective activation of S1PR_1_.

SVD is recognized as being increasingly diverse with apparently different subtypes [72], and no animal models presenting all of the main disease features are, as of yet, available [38, 73, 74]. The SHRSP is a well-accepted model for studying hypertension and cerebrovascular disease [75]. We have previously reported that SHRSPs subjected to UCAO/JPD developed WM lesions (WML) and cognitive impairment as a result of BBB disruption and inflammation, secondary to chronic hypoxic hypoperfusion [35-38]. In addition, this model presented reliable WMLs at 4 weeks after UCAO/JPD at susceptible brain regions [34], an important criteria for animal model use indicated by the AHA Scientific Statement [75]. In this study, SHRSPs showed endothelial injury with microbleeds in the WM at an early stage after UCAO/JPD onset, which is seen in human SVD development [76]. Chronic hypoxia disturbed the polarity of the TJs in cerebral ECs and caused erythrocyte accumulation in the capillaries in SHRSPs [77]. This endothelial dysfunction eventually results in BBB leakage and vessel wall microbleeds [21, 23], contributing to the onset of SVD [22]. Besides WMLs, our MRI images also demonstrated that GM lesions (GML) in the cortex and hippocampus, as seen in SVD patients [78], are related to BBB leakage development in UCAO/JPD SHRSPs. Neuroimaging studies show that higher WM hyperintensity (WMH) burden is correlated with greater microglial activation in human SVD [65]. Progressive increase in microgliosis with increasing pTau (such as AT8) pathology are associated with arteriolosclerosis in human SVD [79, 80]. Our findings in this study demonstrated that the WMLs and GMLs shown by MRI are spatiotemporally correlated with progressive microglia activation and pTau (AT8) accumulation [52], over 4 to 9 weeks after UCAO/JPD onset. Together, our previous and present data provided compelling neuroimaging and neuropathological evidence that UCAO/JPD SHRSP is an adequate model for evaluating the molecular and cellular mechanisms of how hypoxia hypoperfusion induced endothelial dysfunction and BBB disruption like those occurring in SVD.

The primary goal of this study is to determine the impact of chronic hypoxic hypoperfusion on cerebral capillary endothelial S1PR_1_ and its correlation with BBB integrity disruption in UCAO/JPD SHRSPs. To this end, we performed experiments in which we assessed either direct effects of chronic hypoxic hypoperfusion or effects of selective S1PR_1_ agonist on endothelial S1PR_1_ and BBB integrity. S1P is a bioactive sphingolipid that, acting through its five G-protein coupled S1P-receptors (S1PR_1-5_), modulates a large diversity of biological mechanisms, including cell proliferation and survival, cytoskeletal reorganization and migration [24, 25], and BBB integrity [81, 82]. In cerebral vascular ECs, S1PR_1_ is needed for TJ complex assembly and normal function of the BBB by promoting the expression of TJPs and AJPs in ECs and astrocytes (ACs), as well as restricting leukocyte infiltration [27, 83]. Studies have reported that endothelial injury and inflammation lead to degradation of S1PR_1_ [25, 57]. Endothelial-specific *S1pr1* knockout mice displayed protracted BBB leakage, and the chronic BBB leakiness was associated with cognitive impairment, while pharmacological inhibition of S1PR_1_ function led to transient BBB breach [29]. Here, we provide evidence that chronic hypoxic hypoperfusion disrupts cerebral capillary endothelial S1PR_1_, leading to progressive loss of S1PR_1_ in both rat and human brains with SVD pathological changes. Our results also suggest that long-term selective activation of S1PR_1_ significantly protects S1PR_1_ from degradation, resulting in preservation of TJPs, particularly enhancing the expression of claudin-5 in UCAO/JPD SHRSPs. Notably, the Western blot analysis demonstrated that claudin-5 is the TJP mostly hit by the chronic hypoperfusion hypoxia, compared with ZO-1 and occludin, in both WMs and GMs. Claudin-5 was previously found specifically in ECs, in large amounts, especially in the brain ECs [43, 63, 84], while ZO-1 and occludin can also be expressed by reactive glial cells in injured brain [34, 43]. Our results indicate that SEW2871 reverses UCAO/JPD induced claudin-5 degradation in ECs by activating S1PR_1_, leading to enhancement of endothelial TJ formation and microvascular BBB integrity in the lesioned areas. In preliminary dose-range data by DCE-MRI, not shown here, rats treated with SEW2871 (0.5 mg/kg) demonstrate lower BBB permeability. This finding is in concordance with the recent demonstration that endothelial specific-*S1pr1* deletion induced a size-selective BBB opening in mice (3-10 kDa), which is similar to that of *Claudin-5* and *Occludin* double knockdowns [29]. This finding also highlighted S1PR_1_ signalling as a potential novel mechanism that links BBB dysfunction and tissue hypoxic hypoperfusion and underlies the essentials of SVD in the SHRSP/UCAO/JPD model.

Currently, one of the major challenges for SVD research is to determine which vascular dysfunctions are reversible [78]. Cerebral capillary endothelial dysfunction is considered to be a prelude to vascular dementia [22]. Vascular hypertrophy and inward remodeling lead to a progressive reduction of CBF in capillaries [1, 8, 17] and result in chronic tissue hypoxic hypoperfusion [85], which directly damages the BBB integrity [21, 23] and accelerates the neurodegenerative process of SVD [86]. Therefore, we expected that reversal of BBB disruption and capillary dysfunction through pharmacological intervention of S1PR_1_ signalling will be accompanied by reduced SVD pathology. Here we show that rats treated with SEW2871 to activate S1PR_1_ at the beginning of UCAO/JPD onset reduces brain injury in the cortex, WMs, and hippocampus after exposure to UCAO/JPD for 4 weeks, as monitored by T2 weighted MRI. Cerebral microinfarcts are small lesions that are presumed to be ischemic, which commonly occur in patients with stroke and dementia and their contribution to vascular cognitive impairment is increasingly recognized [87]. Recent neuroimaging studies with ASL MRI suggested that cerebral microinfarcts in memory clinic patients are predominantly related to global reductions in cerebral perfusion, in line with other manifestations of SVD [88]. Our ASL MRI analysis further indicated that treatment with SEW2871 significantly improves global CBF in the rat brains subjected to chronic hypoxic hypoperfusion, suggesting a protective role of SEW2871 against hypoperfusion-induced vascular injury. As already discussed, SVD, specifically the arteriolosclerosis pathology, is linked with Tau pathological changes in the aging brain, while Tau tangle pathology has been clinically suggested to modify the association between SVD and cortical microinfarcts in human [79]. UCAO/JPD induced pTau accumulation in the cortex and WMs, where T2 imaging showed hyperintensity, in SHRSP rats. In the cortex, our histological data shows that pTau primarily accumulates in neurons that locate within cellular layers V and VI areas, where neurons are suggested for maintenance and control of working memory [54]. Human studies also show that pTau can also be detected in astrocytes, around the perivascular space, and oligodendroglial coiled bodies in the aging brain [89], which was suggested to be associated with white matter demyelination [90]. In support of the observation in humans, we showed an increased expression of pTau in WM (lesioned corpus callosum) of UCAO/JPD SHRSPs, histologically and biochemically. One of the potential mechanisms linking SVD and tau pathology is that neuroinflammation and BBB dysfunction impact key pathways that regulate intrinsic neuronal/cellular activity and function, thus leading to abnormal protein aggregation and neurodegeneration [80, 90]. By activating S1PR_1_ with SEW2871 to protect the BBB from disruption, we show significant reduction of pTau protein levels in the cortex, WMs, and hippocampus. Further work exploring key molecular mechanisms linked to BBB permeability and pTau accumulation will be important. Taken together, our data demonstrates the potential of the S1P-S1PR_1_ signalling pathway as a protector of vascular function through preventing BBB leakage and the progressive development of SVD. We speculate that interventions aimed at the S1PR_1_ signalling pathways, by promoting restoration of BBB integrity in the microvasculature, could prevent the onset or the progression of SVD.

Despite the importance of S1PR_1_ in maintaining the BBB, the mechanisms by which S1P-S1PR_1_ signalling impacts the BBB are understudied. Generally, S1PR_1_ signalling acts at multiple levels to enhance vascular function [29]: S1PR_1_ exclusively couples with G_i/o_, which may lead to activation of the PI3K/Akt pathway to prevent apoptosis, and PI3K/Rac to promote cytoskeletal reorganization, proliferation, and migration [66, 67]. Indeed, S1PR_1_ regulates vascular permeability through cytoskeletal reorganization, AJ and TJ assembly, and focal EC-to-EC adhesion formation [27, 91]. Akt is known to be critical for cell survival, proliferation, and gene expression. In ECs, S1P signals through the S1PR_1_ to heterotrimeric G protein G_i_, leading to activation of serine/threonine kinase Akt and phosphorylation of the Akt substrate, while Akt activity is required for S1PR_1_ to activate Rac [92, 93]. Western blot analysis for pAkt protein levels in SHRSP rat brain tissues showed that UCAO/JPD caused significant reduction of pAkt in WMs and GMs, which was revealed by treatment with SEW2871, suggesting that S1PR_1_ activation by SEW2871 to prevent BBB integrity and endothelial survival is mediated via the PI3K/Akt pathway. One of the primary pathways affected by G_i_ activation in S1PR_1_ signalling is Erk-dependent proliferation [27]. Interestingly, our data show that UCAO/JPD elevated pErk1/2 protein levels in WM and GM, when S1PR_1_ began to reduce in SHRSP rats. On the other hand, selective activity of S1PR_1_ with SEW2871 alleviates activation of pErk1/2. These atypical changes led us to alternative signalling pathways. A study reported that S1PR_2_ antagonist alleviated oxidative stress-enhanced brain EC permeability by attenuating Erk1/2 pathway [32]. Activation of S1PR_2_ signalling results in Erk1/2 mediated inhibition of serum-induced proliferation [94]. Our preliminary data shows that UCAO/JPD progressively increases S1PR_2_ expression starting from week 4 and reaches statistical significance at week 9, indicating roles of S1PR_2_ in pathological development in UCAO/JPD rat brains. When S1PR_1_ was activated with SEW2871, both S1PR_2_ and pErk1/2 protein levels decreased in WM and GM. These data are in concordance with recent findings that suppressing S1PR_2_ activity attenuates activation of microglia and M1 polarization, along with attenuated activation of its effector Erk1/2 pathway including Erk1/2 in post-ischemic brain [95]. ECs express S1PR_1_, S1PR_2_, and S1PR_3_ proteins [69]. Conversely with S1PR_1_ and S1PR_3_, S1PR_2_ increases vascular permeability, enhances leukocyte entry [26, 27, 30, 31] via Rho/ROCK pathways and inhibits S1PR_1_ [69-71]. S1PR_2_ activity impairs remyelination, enhances BBB leakage, and demyelination in animal models of multiple sclerosis [96]. Our finding is in line with previous reports that indicated an antagonistic relationship between S1PR_1_ and S1PR_2_ in the vascular endothelium during tissue injury and disease [69]. While our data sugest that PI3K/Akt pathway and S1PR_2_/Erk1/2 pathway are associated with the neurovascular protective effect of SEW2871 on S1PR_1_ activation, future work exploring key molecular vascular markers linked to these pathways will be important.

In summary, findings from this study advance our knowledge regarding endothelial S1PR_1_ signalling as a potential molecular mechanistic basis that links the hypoxic hypoperfusion and BBB damage in the neuropathological cascades in SVD. A major strength of this study is that specifically promoting S1P-S1PR_1_ signalling at an early stage preserves the integrity of endothelial junctions and BBB, which could slow down the progression of SVD. In addition, the combined use of pre-clinical MRI with histological and biochemical analyses allows us to obtain further understanding of how cerebral hypoxic hypoperfusion induces the morphological and molecular changes that compromise BBB integrity, leading to WML and GML in the rat brains subjected to UCAO/JPD. However, we recognized several limitations. Despite the fact that our rat model captures multiple pathological aspects of human SVD, a complex disease, experimental animal species differ from humans in many aspects, such as rodents display a minimal amount of brain white matter and have a much shorter lifespan, which is inherently limited for use in SVD research. Since the time course to produce an injury in the SHRSP brain is short, the translational relevance of the molecular and cellular pathways that lead to BBB damage in this model compared to human SVD mechanisms, where SVD develops on a chronic long-term timeline in older patients, needs further multi-disciplinary evaluation [97]. Although we examined the role of SEW2871 on BBB integrity and pTau accumulation, S1PR_1_ is also expressed in other cellular components of the neurovascular unit. Subsequent investigation for the specific effect of SEW2871 on endothelial S1PR_1_ is necessary. As our DAB immunohistological data demonstrated a neuronal loss of S1PR_1_ in human SVD, investigation to examine the additional effects of SEW2871 on SVD markers, such as neuroinflammation, neuronal death, and astrogliosis, is needed. Futhermore, SEW2871 treatment was examined at only one time point (week 4) since this is a proof of principle investigation to determine whether interventions aimed at S1PR_1_ signalling pathways could ameliorate the progression of SVD through restoration of BBB integrity. Finally, due to the preliminary nature of the study, data on SEW2871 dose range evaluations with behavioural tests were not included.

## Data Availability

Data supporting the present study are available from the corresponding author upon reasonable request.
